# American Medical Association

**Published:** 1873-07-01

**Authors:** 


### MEDICAL EXAMINER.

*A Semi-Monthly Journal of Medical Sciences.*

EDITED BY

N. S. DAVIS, M. D., AND F. H. DAVIS, M. D.

Chicago, July 1st, 1873.

### EDITORIAL.

### AMERICAN MEDICAL ASSOCIATION.

WHAT WAS DONE AT THE RECENT ANNUAL MEETING CONCERNING ITS CONSTITUTION AND BY-LAWS.

Most of our exchanges have published the record of proceedings of the recent annual meeting of the American Medical Association so imperfectly and erroneously, that we have thought it proper to give a corrected statement of what was actually done in reference to improvements in the organization and practical working of the Association. Such caricatures as were published in the *Clinic*, of Cincinnati, over the significant cognomen of *John Smith, M.D.*, and copied by the Chicago *Medical Journal*, and partly by the Leavenworth *Herald*, are too contemptible to merit even a passing notice. But such periodicals as the *Medical Record*, the Philadelphia *Medical Times*, the Boston *Medical and Surgical Journal*, etc., should have sufficient regard for the interests of their readers to ensure a reasonable degree of correctness in stating the doings of the National organization. While the editors of most of these periodicals have remained at home, nursing their chronic habit of fault-finding, a few active-working members of the Association have been patiently endeavoring to keep out subjects of strife and contention; to increase the efficiency of the practical working of the Sections; and to make such improvements in the general organization, from time to time, as experience should dictate. To secure the first of these objects, in the future, the recent meeting of the Association adopted, unanimously, the following additional by-laws:

*XI. Judicial Council.*—A Council, consisting of twenty-one members, shall be appointed by the Nominating Committee, whose duty it shall be to take cognizance of and decide all questions of an ethical or judical character that may arise in connection with the Association. Of the twenty-one members of the Council first appointed, the seven first named in the list shall hold office one year, and the second seven named shall hold office two years.

With these exceptions the term of office of members of the Council shall be three years, seven being appointed by the Nominating Committee annually.

The said Council shall organize by choosing a President and Secretary, and shall keep a permanent record of its proceedings. The decisions of said Council on all matters referred to it by the Association shall be final, and shall be reported to the Association at the earliest practicable moment.

All questions of a personal character, including complaints and protests, and all questions on credentials, shall be referred at once, after the report of the Committee of Arrangements, or other presentation, to the *Judicial Council*, and without discussion.

To improve the practical working of the Sections, and add to the scientific value of the general meetings, the following regulations were adopted with great unanimity:

*Resolved*, That Sec. 9, Art. I. of the By-laws be amended to read as follows: "The reading and consideration of the reports of the Standing Committees of Publication and on Prize Essays, and of Chairmen of Sections."

*Resolved*, That Art. II. of the By-laws be amended as follows: In first paragraph strike out order of Sections as it now stands and insert instead,

"1. Practical Medicine, Materia Medica, and Physiology.

2. Obstetrics, and Diseases of Women and children.

3. Surgery and Anatomy.

4. Medical Jurisprudence, Chemistry, and Psychology.

5. State Medicine and Public Hygiene."

After second paragraph insert, "The Section on State Medicine and Public Hygiene shall be composed of one member from each State, representing, as far as practicable, the State Boards of Health. The officers of this Section to be also designated by the Committee on Nominations." "The Chairman of the several Sections shall prepare and read in the general sessions of the Association, papers on the advances and discoveries of the past year in the branches of science included in their respective Sections; the reading of such papers not to occupy longer than forty minutes for each."

After third paragraph insert, "No paper shall be read before either of the Sections the reading of which occupies more than twenty minutes. Such papers shall be referred by the Section to sub-committees especially appointed for their examination. The sub-committees shall be allowed thirty days for such examination, at the end of which time they shall forward the papers to the Committee of Publication, with such recommendation as they may deem proper. The authors of such papers, however, may read abstracts before the Section, within the allotted twenty minutes. No member shall address the Section more than once on the same subject, nor speak longer than fifteen minutes, without unanimous consent."

*Resolved*, That Art. III. of the By-laws be amended as follows: Strike out all that relates to the Committees on Medical Education, on Medical Literature, and on Climatology and Epidemic Diseases.

*Resolved*, That Art. IV. of the By-laws be amended by adding, "The Committee of Publication shall have full discretionary power to omit from the published transactions, in part or in whole, any paper that may be referred to it by the Association, or either of the Sections, unless specially instructed to the contrary by vote of the Association."

*Resolved*, That a new Section—XII.—be added to the By-laws, as follows: "No new business, resolutions by members, etc., shall be introduced at the general session of the Association, except on the first and fourth days of meetings."

1. *Resolved*, That the Committee of Arrangements for each annual meeting of the Association are instructed to secure the services of a sufficient number of stenographic reporters to have one in attendance on the regular sessions of each of the Sections in the afternoon, as well as during the General morning sessions. That the reports thus obtained be printed, the same evening, on slips of proof-sheets, in sufficient number to supply all the members of the Association in attendance, early the following morning.

2. *Resolved*, That the necessary expenses incurred in carrying into effect the foregoing resolution shall be paid from the Treasury of the Association, in the same manner as other bills relating to the publication of the Transactions.

3. *Resolved*, That the acting Secretary of each *Section* shall keep a file of so much of the reports as relate to the business of the Section to which he belongs; and it shall be his duty to revise the same, carefully eliminating therefrom all that is unimportant, and arranging that which is found valuable and pertinent in proper form; he shall transmit the same to the Permanent Secretary for the Committee of Publication within twenty days after the adjournment of each annual session of the Association.

Whoever will take the trouble to compare the foregoing amendments and additions to the By-laws with the previous provisions, and their practical working, will readily perceive that they are designed to accomplish, first, a more efficient organization of the *Sections*, with more thorough examination, by sub-committees, of all papers presented, and full reports of their discussions; second, the presentation, in the general sessions, of an address on the progress of each of the important departments of medicine by the Presidents of the Sections, instead of annually repeated reports on Medical Education, Literature, etc.; and, third, the reference of all more personal, ethical in judicial matters, directly to a permanently organized and competent "Judicial Council." If the fault-finders, as well as the active friends of the Association, will study the provisions adopted, and aid in carrying them into practical operation, they will find little to complain of in the future meetings.

When the Association was first organized there were but few State Medical Societies in active existence, and it was thought best to provide for delegates from all regular medical organizations, whether societies, schools, hospitals or asylums; the object being to unite all the interest of the profession in the national organization. Such a course was undoubtedly wise at that time. But the door thus widely opened has led to certain abuses which need correction; while the regular State, County and District Societies have multiplied until they exist in almost every State and Territory in the Union.

Hence, it becomes an important question, which should be carefully considered, whether representation in the Association ought not hereafter to be restricted to such Societies. To bring this question to a practical issue, the following amendments to the Constitution were offered, and will be ready for final action at the next meeting:

Dr. N. S. Davis, of Illinois, offered the following, which was laid over until next session:

It is proposed to strike out the second paragraph of Article II., commencing with the words, "*The Delegate*," and ending with "of Paris," and insert the following:

"The delegates shall receive their appointment from permanently organized *State* Medical Societies, and such County and District Medical Societies as are recognized by representation in their respective State Societies, and from the Medical Department of the Army and Navy of the United States."

Also, strike out all of the fourth paragraph of same Article II., beginning with "Each local Society," and ending with "one delegate," and insert the following:

"Each State, County, and District Medical Society, entitled to representation, shall have the privilege of sending to the Association one delegate for every ten of its regular resident members, and one for every additional fraction of more than half that number.

"The Medical Staffs of the Army and Navy shall be entitled to four delegates each."

